# Establishment and characterization of primary epithelial cell cultures from healthy canine mammary gland tissue

**DOI:** 10.3389/fvets.2025.1652991

**Published:** 2026-01-28

**Authors:** Natalia Nosalova, Mykhailo Huniadi, Alexanda Valencakova, Lubica Hornakova, Blazej Kalinaj, Viera Almasiova, Dana Marcincakova, Peter Kubatka, Slavomir Hornak, Dasa Cizkova

**Affiliations:** 1 Small Animal Clinic, University of Veterinary Medicine and Pharmacy in Košice, Košice, Slovakia; 2 Department of Anatomy, Histology and Physiology, University of Veterinary Medicine and Pharmacy in Košice, Košice, Slovakia; 3 Department of Pharmacology and Toxicology, University of Veterinary Medicine and Pharmacy in Košice, Košice, Slovakia; 4 Institute of Neuroimmunology, SAS, Bratislava, Slovakia

**Keywords:** biomarkers, canine mammary gland, histology, isolation of cells, oncology, primary cell cultures

## Abstract

**Introduction:**

In regenerative medicine and comparative oncology, the development of physiologically relevant in vitro models is critical for advancing our understanding of tissue homeostasis, cellular differentiation, and early tumorigenesis. Such models provide controlled experimental systems to investigate normal mammary gland function, assess responses to therapeutic agents, and establish baseline characteristics for distinguishing healthy from pathological tissue.

**Methods:**

This study successfully established and characterized primary epithelial cell cultures derived from histologically normal canine mammary gland (CMG) tissue. Samples from two healthy female dogs were obtained during elective ovariohysterectomy. Following enzymatic digestion and optimized culture conditions, isolated cells adhered within 24 hours and reached confluence within 6–7 days, maintaining over 95% viability.

**Results and discussion:**

Histological analysis confirmed either active lactational or regressive tissue states. Cell growth and metabolic activity were evaluated using the CELLigence system and XTT assay, with optimal results achieved at a seeding density of 4,000 cells per well. Immunofluorescence staining confirmed epithelial identity (pan-CK, CK8/18), apical MUC1 expression, low Ki-67 levels and negative expression of mesenchymal markers (vimentin, S100), indicating a healthy, non-cancerous cell population with moderate proliferative activity. This study provides the first detailed protocol for establishing primary CMG epithelial cultures and validates their suitability as a reference model for future oncological studies. Overall, this platform supports translational research, including drug testing and biomarker discovery, contributes to personalized veterinary therapies and enhanced understanding of mammary gland pathology in both dogs and humans.

## 1 Introduction

The active mammary gland is a compound, tubuloalveolar gland found only in mammals, designed to feed the offspring and support its immune system (1). Glandular parenchyma consists of alveoli and ductal structures organized into lobules, which are surrounded by connective tissue septa. The ductal system begins with an intralobular duct, which drains into an interlobular duct and finally into lactiferous ducts. Several lactiferous ducts drain into a lactiferous sinus, which joins the teat sinus (2). The mammary gland is highly dependent on hormonal stimulation, and its development and action are controlled by various hormones (prolactin, estrogen, and progesterone). The mammary gland is also strongly influenced by the estrous cycle and pregnancy (1). The mammary gland of women and dogs share similar morphological characteristics, differing in the number of glandular units, number of nipples/teats, hormonal regulation, functional cycles and milk composition. In dogs, four to six pairs of glands have been described, with the inguinal pairs being the largest, the abdominal pairs intermediate, and the thoracic pairs the smallest in size (1).

Due to the constantly increasing number of cases and the clinical significance of breast cancer (BC), research into physiology and pathology of mammary glands has increased significantly. In this context, female dogs have emerged as valuable comparative animal models for studying human cancer, providing samples for the development of alternative *in vitro* models and playing a key role in advancing progress in this research area (3). Building on tissue-derived cell cultures, established cell lines play a fundamental role in research for their importance in the study of diseases, stem and cancer cells investigation, drug discovery, and the establishment of new therapies (4). Even though immortalized cell lines are widely employed in biomedical research because of their reproducibility and ease of maintenance, they often differ greatly from *in vivo* cells, exhibiting altered gene expression profiles and the loss of tissue-specific functions (5). For these reasons, primary cell cultures isolated from tissues are becoming more widely acknowledged as more physiologically relevant models in molecular biology, oncology and pharmacological research (6). Furthermore, they serve as an excellent preclinical model essential for studying gene expression, modification of signaling pathways, oncogene activation, and the development of new drugs and therapeutic strategies (7). Several cell types can be isolated from the canine mammary gland, including epithelial cells, myoepithelial cells, stromal fibroblasts, adipocytes, mesenchymal stem cells (MSCs), and resident immune cells (1, 3, 4, 7, 8). Among these, primary cultures of mammary epithelial cells are the most commonly used in research because of their importance in studying gland development, hormonal regulation, lactation physiology, and neoplastic transformation (4, 8). These cultures serve as essential reference models for investigating basic physiological processes such as proliferation, hormonal interactions and differentiation. In the context of cancer research, they allow for direct comparison with tumor cells, assisting to identify cancer specific alterations. Additionally, they are invaluable for assessing treatment methods and play a key role in the development and validation of diagnostic and prognostic markers for canine mammary tumors (8, 9).

In line with this, the present study aimed to establish and characterize primary epithelial cell cultures from healthy canine mammary gland (CMG) tissue as a relevant *in vitro* model for studying normal mammary biology and early neoplastic transformation. Key objectives included: confirming the physiological status of donor tissue via histology; assessing cell morphology, adherence, and proliferation using xCELLigence and XTT assays; and verifying epithelial identity through immunofluorescence staining (pan-CK, CK8/18, MUC1, Ki-67). By optimizing growth conditions and validating cell phenotypes, the study provides a foundation for developing standardized cell lines to support comparative oncology and personalized veterinary therapies.

## 2 Materials and methods

### 2.1 Animals and tissue samples

At the beginning of the experiment, informed consent was obtained from the owners of the animals to collect biological material. The research protocols were approved by the ethics committee of the UVMP in Košice (EKVP/2023–02). The tissue donors were two female dogs, an 8-year-old French Bulldog (C1) with a weight of approximately 9 kg and a 1-year-old Dachshund (C2) with a weight of 7.7 kg. The collection of healthy mammary gland tissue (n = 2) was performed during preventive ovariohysterectomy at Small Animal Clinic UVMP in Košice according to the standard veterinary procedures under strict sterile conditions. After surgical excision, the tissue sample (approx. 1 cm^3^) was transported to the laboratory in a sterile solution consisting of 1x phosphate-buffered saline (PBS, Sigma, United States) supplemented with 0.02% gentamicin solution (50 mg/mL, Sigma, USA). The tissue was mechanically divided into smaller pieces under sterile conditions. A portion of the tissue was transferred into PBS containing gentamicin (0.02%) for subsequent cell isolation. Smaller fragments of glandular tissue (approx. 0.5 cm^3^) were immediately fixed in 4% formaldehyde to preserve the tissue architecture for histological and immunohistochemical analysis.

### 2.2 Isolation of primary cell cultures

The isolation of primary cell cultures was performed in a Biosafety Cabinets (Herasafe, Class II Biological Safety Cabinet, Thermo Fisher Scientific Inc., United States, BioSafe, Class II. A2, Ekokrok, SK) under sterile conditions to prevent contamination by unwanted pathogens. The canine mammary gland tissue immersed in PBS was first mechanically dissociated using surgical instruments. Then enzymatic digestion was performed with 1 mg/mL collagenase IV (Gibco, Invitrogen, Carlsbad, CA, United States) in Earle’s Balanced Salts Solution (Biosera, France) supplemented by 1% Antibiotic/Antimycotic solution (ATB/ATM, 1% solution of penicillin (10,000 U/mL), streptomycin (10,000 μg/mL), and amphotericin B (25 μg/mL)Sigma, United States) and 0.1% gentamicin. After 45 min of incubation at 37 °C, the enzyme was neutralized with the same volume of fetal bovine serum (FBS, Sigma, United States). The digested tissue was filtered through a cell strainer with the size of 100 μL and centrifuged at 1200 rpm/ 7 min. The cell pellet was resuspended in 1 mL of culture medium Dulbecco’s modified Eagle’s medium High Glucose (DMEM HG, Sigma, CA, United States). The cells were plated on a 25 cm^2^ tissue culture flask/T25 (Corning, Lowell, MA, United States) and maintained in a humidified atmosphere containing 5% CO_2_ at 37 °C.

### 2.3 Morphology, cultivation, and cryopreservation of primary cell cultures

CMG cells were cultivated in DMEM HG supplemented with 10% FBS, 2% ATB/ATM solution, 0.1% gentamicin, and 1% L-glutamine (Sigma, United States) under standard culture conditions (5% CO_2_ at 37 °C) until the formation of a monolayer. The medium was changed every 2–3 days of incubation. The adherence properties, morphology and expansion capacity of CMG cells were evaluated after 24, 48, and 72 h of cultivation using the inverted microscope Zeiss Axiovert 200 equipped with a digital acquisition system. When the cell confluence reached 80–90%, primary culture cells were subcultured to establish passage 1 (P1). The CMG cells were washed with PBS and detached from the bottom of the culture flask using 1 mL of 0.05% trypsin/EDTA solution (Sigma, CA, United States). After incubation for 3–5 min at 37 °C, the enzyme was deactivated with the same volume of FBS. The suspension of cells was centrifuged at 1200 rpm/ 7 min. The supernatant was removed, and 1 mL of DMEM HG was added for resuspension of the cell pellet. Afterward, the cells were counted in a trypan blue 4% solution using a hemocytometer under an inverted microscope. For the counting of the total number of cells, the calculation formula was used: Total number of cells/ml = (Number of viable cells × dilution factor×10^4^)/(Number of squares counted). The percentage of viable cells was calculated as (the number of viable cells / total number of cells) × 100. The part of the pellet was plated on a 75 cm^2^ tissue culture flask/T75 cm^2^ (Corning, Lowell, MA, United States) for further experiments. The rest of the cell pellet was centrifuged (1,200 rpm/7 min) and resuspended in freezing medium composed of 50% DMEM HG, 40% FBS and 10% dimethyl sulfoxide (DMSO, Sigma, United States). The cells in cryogenic vials (Ratiolab, Deutschland) were frozen at 1 °C/min in MrFrosty Freezing Container (Sigma, USA) in 80 °C for 24 h and subsequently stored in liquid nitrogen.

### 2.4 Growth assay

The growth curve of cultured cells was evaluated using the xCELLigence system, Real-Time Cell Analyzer (RTCA, Roche Applied Sciences, Mannheim, Germany) and XTT assay.

The RTCA is a system that relies on electronic detection to monitor biological processes. The RTCA uses E-plates (Acea Bioscience, San Diego, CA, United States) equipped with gold microelectrodes on the surface of the culture plate. These electrodes measure electrical impedance, which allows the system to detect changes in cell properties, such as their adherence, changes in morphology, or proliferation (10). All the changes are expressed as cell index (CI) and recorded in curves. The CMG cells at passage 1 (P1) were seeded into 96-well E-plates at different concentrations of cells, 1 × 10^3^, 2 × 10^3^, 4 × 10^3^, and 8 × 10^3^ cells per well in 200 μL of DMEM HG supplemented with 2% ATB/ATM solution, in triplicate, and cultured at 37 °C and 5% CO_2_ for a total of 100 h. The results were experimentally evaluated following the manufacturer’s instructions for the xCELLigence Real-Time Cell Analyzer (RTCA, ACEA Biosciences, United States).

XTT assay is used for the detection of viable, metabolically active cells, which can reduce yellow XTT dye into orange formazan. The cells (P1) were seeded in a 96-well plate at the same concentrations as those used for the xCELLigence assay, in 100 μL of DMEM HG. After three incubation times (24, 48, and 72 h), 50 μL of freshly prepared XTT solution, composed of XTT labeling reagent and electron coupling reagent in a ratio 50:1, was added to the wells. The plates were incubated at 37 °C and 5% CO_2_ for 4 h. The absorbance was measured at a wavelength of 450 nm using the APOLLO Absorbance Reader (Berthold Systems, United States) to analyze cell viability.

### 2.5 Sample preparation for light microscopy evaluation

The tissue samples were immersed in 4% formaldehyde, then dehydrated through a graded series of ethanol (50, 70, 100%), transferred to xylene, and embedded in Paraplast blocks (Sigma-Aldrich). Serial sections (*n* = 6), 6 μm thick obtained from each block were stripped of paraplast and stained using a routine hematoxylin and eosin (H&E) method. Sections were evaluated using a Zeiss Axio Lab A1 light microscope and photodocumented using an Axio Cam ERc 5 camera. Twenty (11) randomly selected fields of each tissue section were evaluated by two independent histologists.

### 2.6 Immunocytochemistry

CMG cells (P2) were seeded on round coverslips (diameter = 12 mm) placed in 24-well plates (1.5 × 10^4^ cells / well) in a final volume of 500 μL. After reaching confluency (4 days), cells were washed with PBS and fixed with 4% paraformaldehyde (pH = 7.2) for 20 min at room temperature. Following fixation, CMG cells were washed again with PBS and incubated in a solution of 10% normal goat serum (NGS) in PBS supplemented with 0.2% Triton X-100 (PBS TX100) for 1 h at room temperature. This process ensures better access of the antibody to intracellular proteins and minimizes non-specific binding. Primary antibodies (Table 1) diluted in PBS TX100 were added to the cells for 24 h at 4 °C. The following day, CMG cells were washed with PBS and incubated with the appropriate secondary antibodies (Table 1) diluted in PBS TX100 for 1 h at room temperature in the dark. During this step, DAPI (1:200) was also added to the cells to stain nuclei. Lastly, cells were washed with PBS three times for 5 min. Coverslips were mounted to the slides using Vectashield mounting medium (Cole-Parmer, Vernon Hills, IL, United States) and analyzed by a Zeiss AxioVision-APOTOM fluorescence microscope. Negative controls were prepared by excluding the primary antibodies from the staining procedure.

**Table 1:** List of antibodies used for immunofluorescence staining of cells and tissues.

Primary antibodies			
Name	Catalog number	Dilution	Manufacturer
Ki-67, RBX-500 UL	ab15580	1:300	Abcam
Mouse Cytokeratin, pan mAb (AE-1/AE-3)	NBP2-29429	1:100	Biotechne
Mouse mAb anti-Cytokeratin 8/18 (5D 3)	NB120-17139	1:100	
Mouse anti-MUC1 (OTI2E3)	NBP2-45838	1:200	
Flex Polyclonal Rabbit Anti-S100	IR504	1:200	Dako
Vimentin Monoclonal Antibody (V9)	MA5-11883	1:250	Invitrogen

Secondary antibodies			
Name	Catalog number	Dilution	Manufacturer
Alexa Fluor™ 488 Goat anti-Rabbit IgG (H + L)	A-11034	1:200	Invitrogen
Alexa Fluor™ 488 Goat anti-Mouse IgG (H + L)	A-11001	1:200	
Alexa Fluor™ 594 Goat anti-Mouse IgG (H + L)	A-11032	1:200	
Alexa Fluor™ 594 Goat anti-Rabbit IgG (H + L)	A-11037	1:200	

### 2.7 Immunohistochemistry

For immunohistochemistry, samples were prepared similarly to the (H&E) staining. The sections were stripped of paraplast and antigen retrieval was carried out with 10 mM citrate buffer (pH = 6) for 10 min at 97 °C, followed by washing with PBS. Then, incubation with 10% NGS, primary and secondary antibodies (Table 1) was performed by the same methodological procedure as for immunocytochemical staining described above. Vectashield medium was used to mount coverslips to the slides. The stained sections were evaluated using a Zeiss AxioVision-APOTOM fluorescence microscope.

## 3 Results

### 3.1 General features of primary canine mammary gland cells

**Figure 1:**
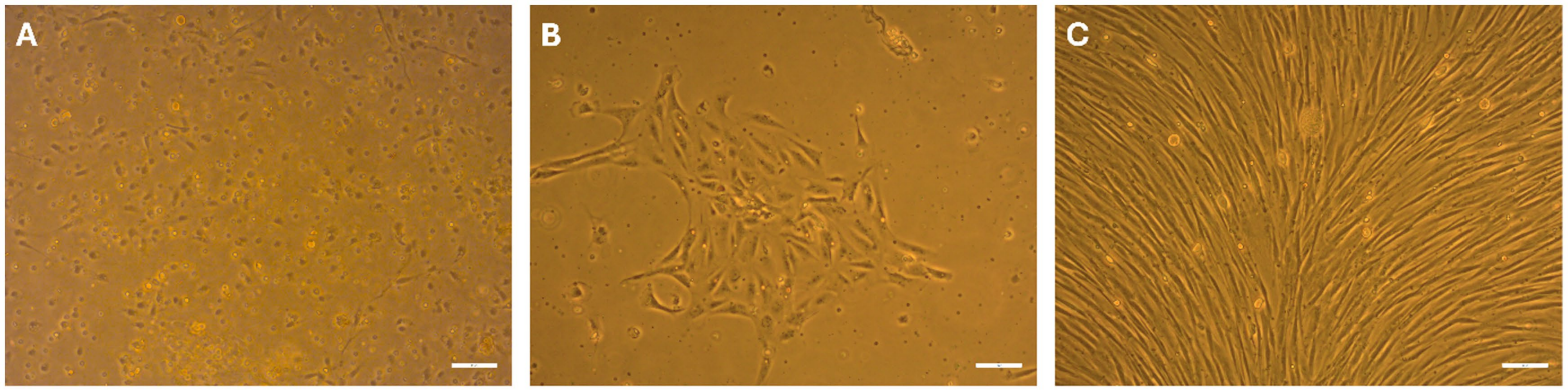
Morphological development of primary CMG cells. Phase-contrast images show CMG cells at passage 0 (P0) on day *in vitro* (DIV) 1 **(A)**, DIV 2 **(B)**, and DIV 7 **(C)**. Cells exhibited initial adherence and polygonal morphology by DIV 1, progressing to fibroblast-like shape (DIV 2) with increased confluence and organization into a monolayer by DIV 7. Scale bars: 50 μm.

With the protocol established herein, primary cells from canine mammary gland tissue were isolated by enzymatic digestion and plated in a culture flask (T25) under standard conditions. The yield of isolated cells was approximately 1 × 10^6^ cells/mL. By the following day, part of the cells had already adhered and changed their shape to a polygonal one. Some rounded precursor cells with light cytoplasm were still actively divided (Figure 1A). At 48 h post-plating, the first medium change was performed, and the cells showed a good attachment to the surface, displaying polygonal and fibroblast-like shapes (Figure 1B). The non-attached material, composed of degenerated cells and detritus, was washed away. After 6–7 days, the monolayers were confluent (Figure 1C), and the CMG cells were subcultured without losing their proliferative properties. Cell viability before each experiment was greater than 95%.

### 3.2 Histological evaluation of canine mammary gland tissue

**Figure 2:**
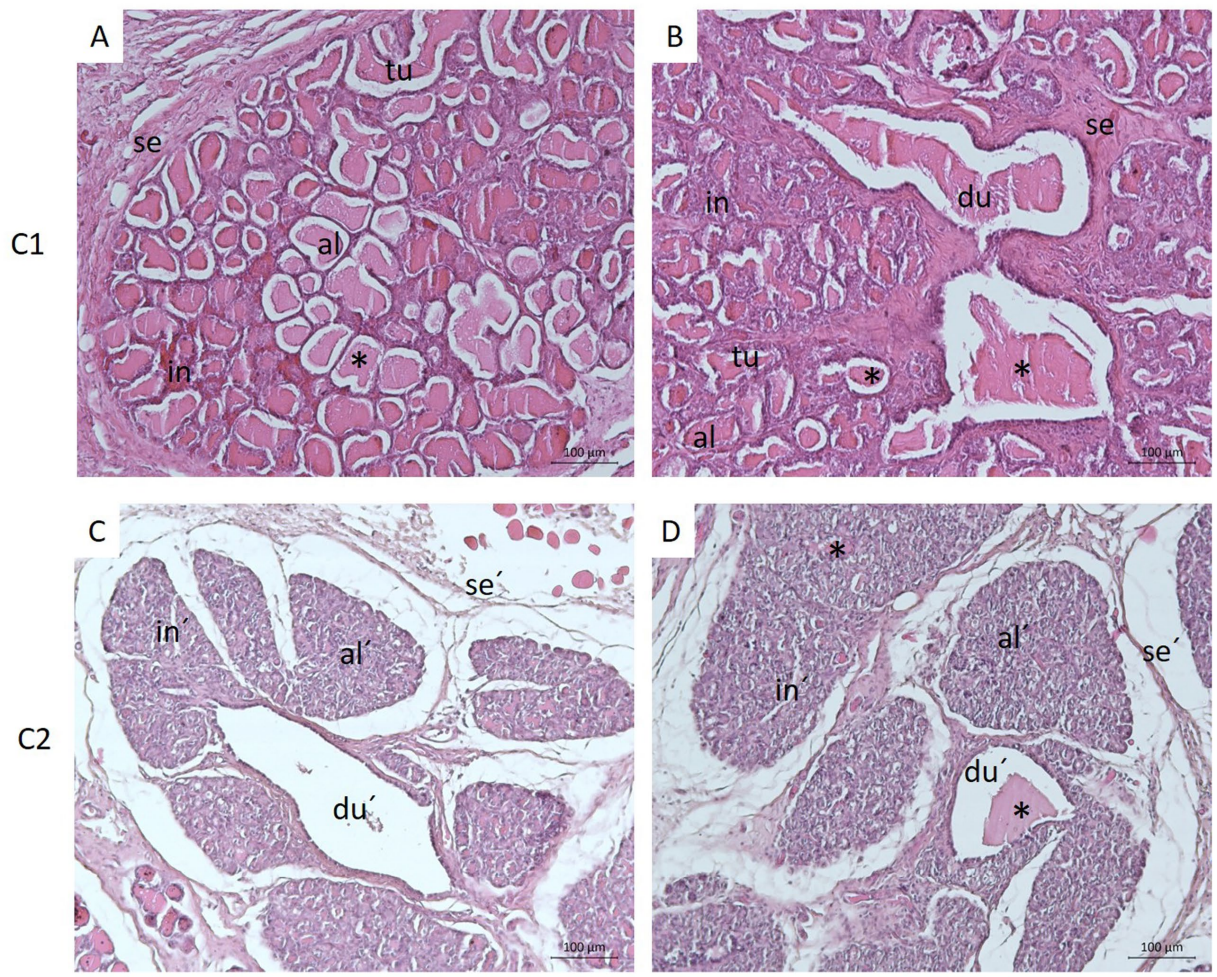
Histological structure of the healthy canine mammary gland during active lactation **(A,B)** and regression **(C,D)**. Panels **A** and **B** show well-developed alveoli (al) and tubules (tu) organized within lobules separated by connective tissue septa (se). Alveoli, tubules, and ducts (du) contained secretory product - milk (*). Panels **C** and **D** illustrate regressive changes of the glandular tissue manifested by collapsed alveoli (al′), distended interlobular ducts (du′) with only occasional occurrence of secretion/milk (*), and expanded connective tissue septa (se´) and interstitium (in´). Scale bar: 100 μm, H&E staining.

The mammary gland of the female dog (C1) (French Bulldog breed) during active lactation consisted of large lobules separated by connective tissue septa. Each lobule contained very well-developed secretory units, alveoli and tubules, which were surrounded by a sparse loose connective tissue-interstitium with small blood vessels. Alveoli, ranging from spherical to ovoid shape and elongated tubules were lined with tall, simple cuboidal secretory epithelium. Lactiferous content was observed in the lumens of both tubules and alveoli. Within the lobules, small intralobular ducts lined by low, simple cuboidal epithelium were found. Septa separating individual lobules contained very well-developed interlobular ducts with large, irregular lumens filled with secretory product/milk (Figures 2A,B).

In the second dog (C2) (Dachshund), the mammary gland was in regression state, and its secretory alveoli and tubules were irregularly shaped, shrunken, and often with completely collapsed lumens. Some alveoli still contained secretory product/milk. The interlobular ducts were highly distended and lined with low, simple cuboidal epithelium. Secretory product was not always found in their lumens. The connective tissue stroma was enlarged (Figures 2C,D).

### 3.3 Growth curve

#### 3.3.1 Proliferation dynamics assessed by real-time monitoring (xCELLigence system)

**Figure 3:**
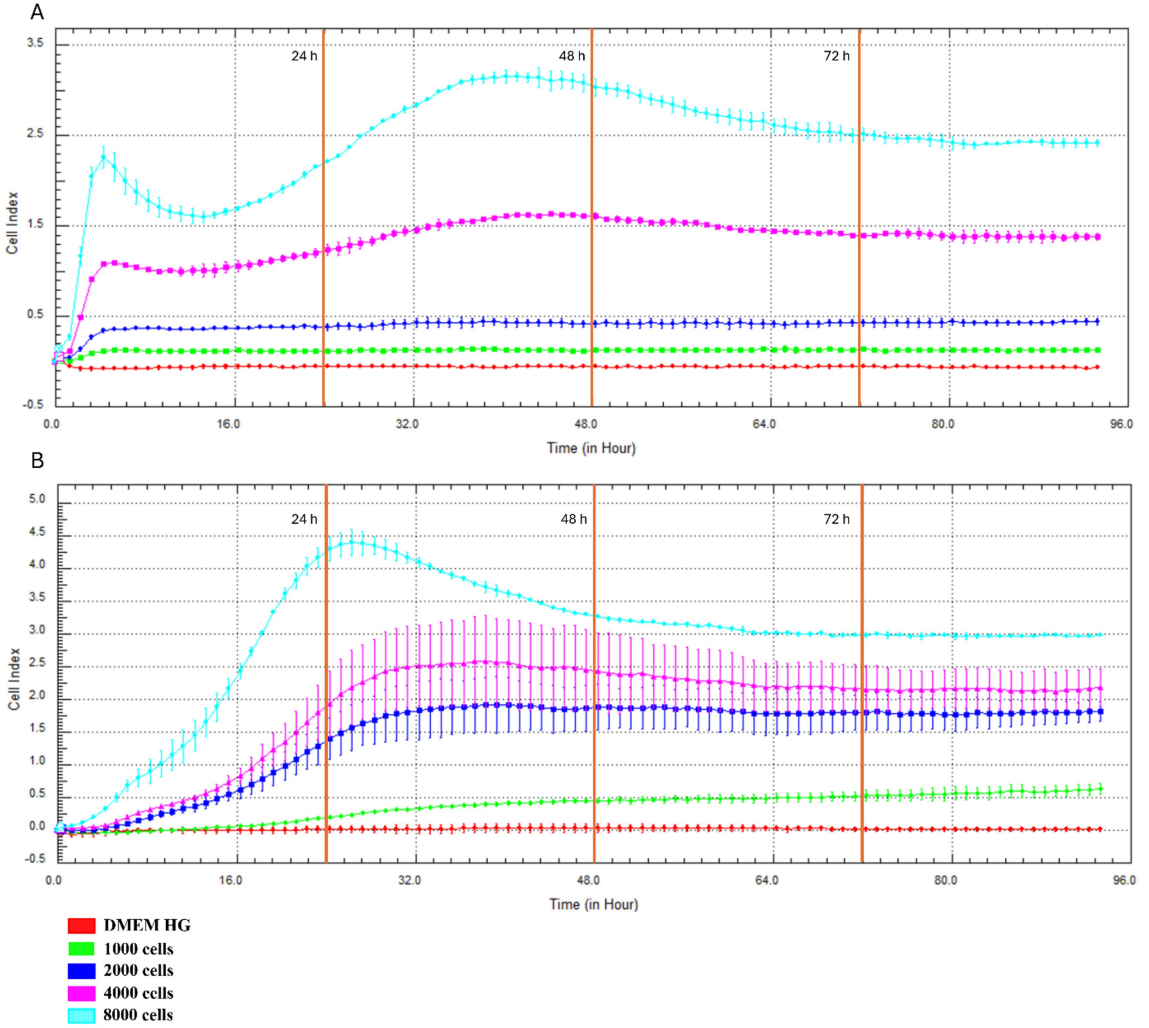
Real-time monitoring of CMG cell proliferation using the xCELLigence system. Growth curves of CMG cell populations C1 **(A)** and C2 **(B)** seeded at 1,000–8,000 cells/well.

The proliferation behavior of primary CMG cells (P1) was evaluated in real time using the xCELLigence system (RTCA), which quantifies the CI as a composite measure of cell number, size, and adhesion. Both CMG cell populations—C1 and C2—demonstrated rapid growth at initial seeding densities of 4,000 and 8,000 cells/well (Figures 3A,B). For C2, increased proliferation was also observed at 2,000 cells/well, though to a lesser extent.

After 48 h, cells at higher densities began to plateau, indicating the onset of contact inhibition. Cultures seeded at 8,000 cells/well reached 80–90% confluence by this time point, after which the CI values slightly declined—suggesting spatial limitations and reduced proliferative activity. In contrast, cells seeded at 4,000 cells/well maintained steady growth between 24 and 72 h, indicating this density as optimal for further *in vitro* experimentation.

#### 3.3.2 Metabolic activity assessment by XTT assay

**Figure 4:**
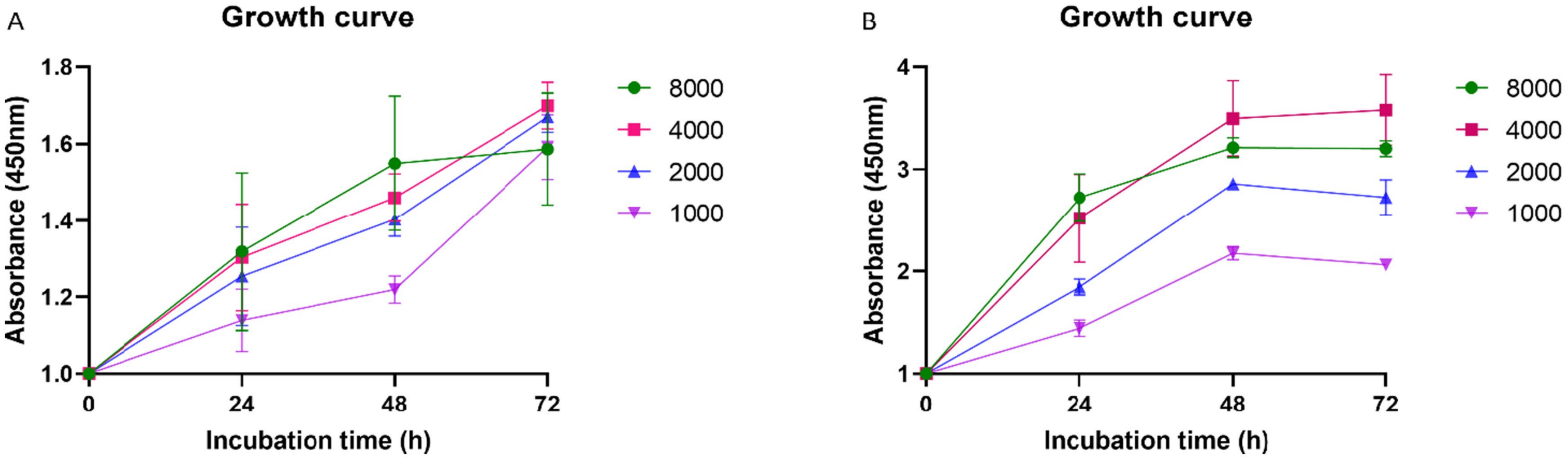
Metabolic activity of CMG cells assessed by XTT assay. CMG cell populations C1 **(A)** and C2 **(B)** were plated at 1000–8000 cells/well under identical conditions to the xCELLigence assay.

To complement real-time proliferation data, metabolic activity of CMG cells (P1) was measured using the XTT assay under identical seeding conditions. As shown in Figures 4A,B, cells seeded at 8,000 cells/well exhibited high metabolic activity within the first 48 h, correlating with the observed early confluence. However, this activity declined thereafter.

Notably, the highest and most sustained metabolic activity over time was recorded in cultures seeded at 4,000 cells/well, corroborating the xCELLigence findings. This concentration supported both robust proliferation and sustained viability, thereby confirming it as the optimal initial seeding density for primary CMG cell culture.

### 3.4 Immunofluorescence analysis of canine mammary gland cells

**Figure 5:**
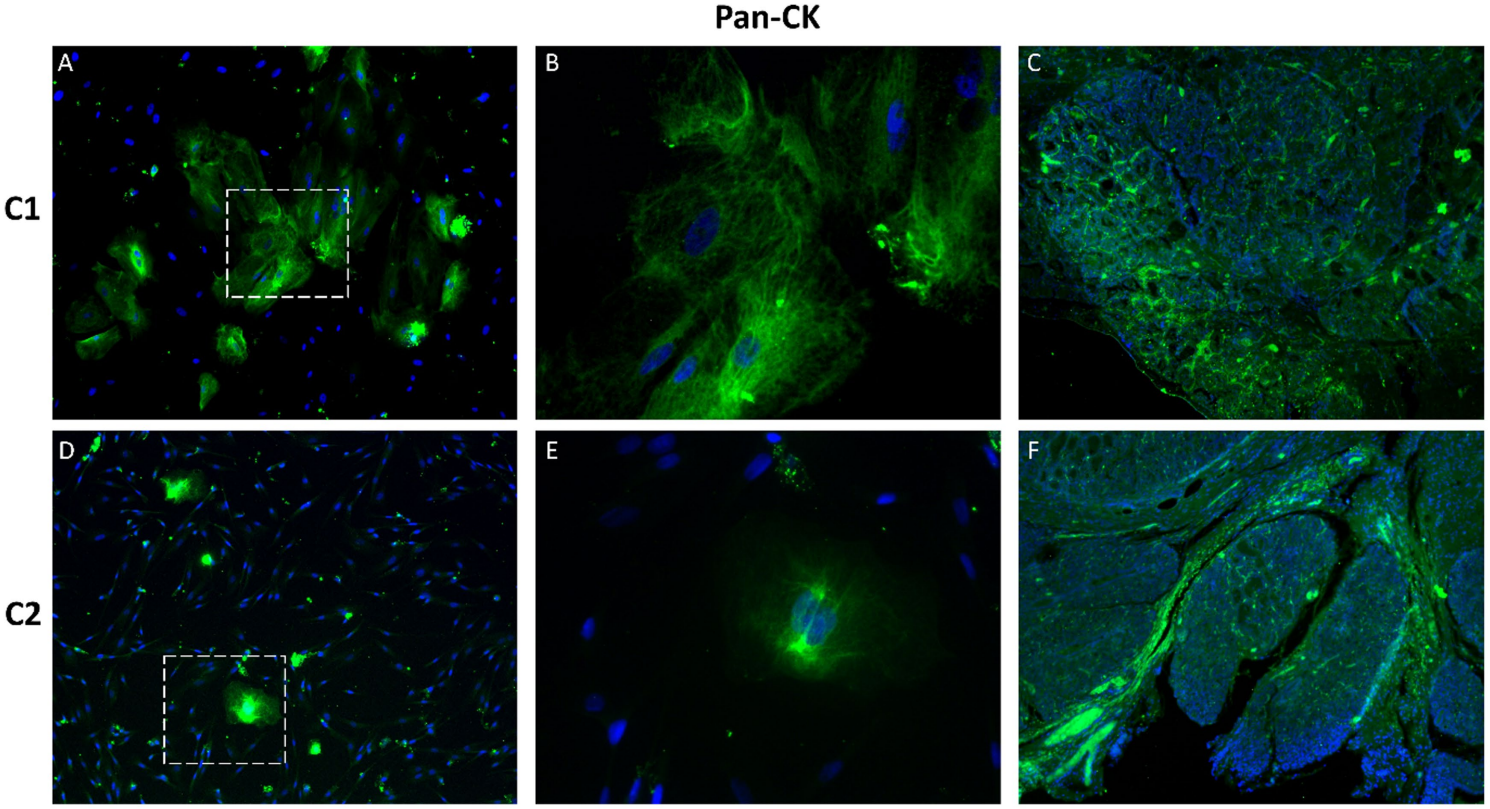
Immunofluorescence staining of pan-cytokeratin (pan-CK) in primary CMG cells and tissue. Pan-CK (green) expression confirmed the epithelial identity of CMG cells in culture **(A,B,D,E)** and tissue sections **(C,F)**. Nuclei are stained with DAPI (blue). **B** and **E** show magnified regions of **A** and **D**, respectively. Magnification: A, C, D, *F* = 100x; B, E = 400x.

**Figure 6:**
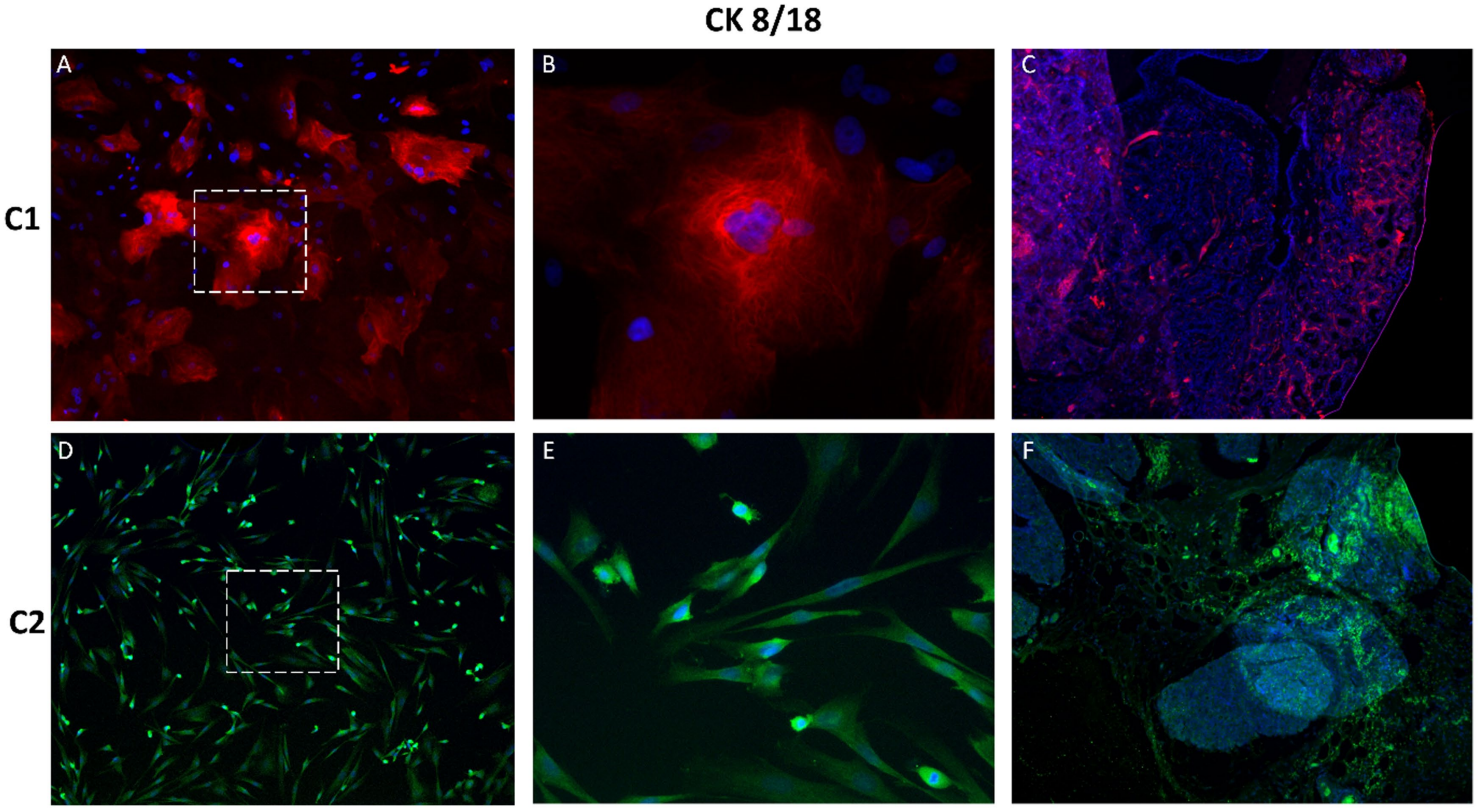
Immunofluorescence staining of cytokeratin 8/18 (CK8/18) in primary CMG cells and tissue. CK8/18 expression was observed in the cytoplasm of CMG cells **(A,B,D,E)** and corresponding mammary tissue **(C,F)**, confirming luminal epithelial identity. Signals were visualized in red or green, depending on antibody labeling. Nuclei were counterstained with DAPI (blue). Panels **B** and **E** show magnified views of **A** and **D**, respectively. Magnification: A, C, D, F = 100x; B, E = 400x.

Immunofluorescence staining was performed to assess the expression of epithelial, mesenchymal and proliferation markers in primary CMG cells (P2) and corresponding tissue samples. The evaluated markers included pan-cytokeratin (pan-CK), cytokeratin 8/18 (CK8/18), mucin-1 (MUC1), Ki-67, vimentin and S100. Strong cytoplasmic positivity for pan-CK and CK8/18 was observed in cultured CMG cells, confirming their epithelial origin (Figures 5, 6). These cytokeratins, which are structural components of the intermediate filament cytoskeleton, are widely used to identify luminal epithelial cells in both normal and neoplastic mammary tissue. Expression patterns in cultured cells closely resembled those observed in native mammary gland tissue.

**Figure 7:**
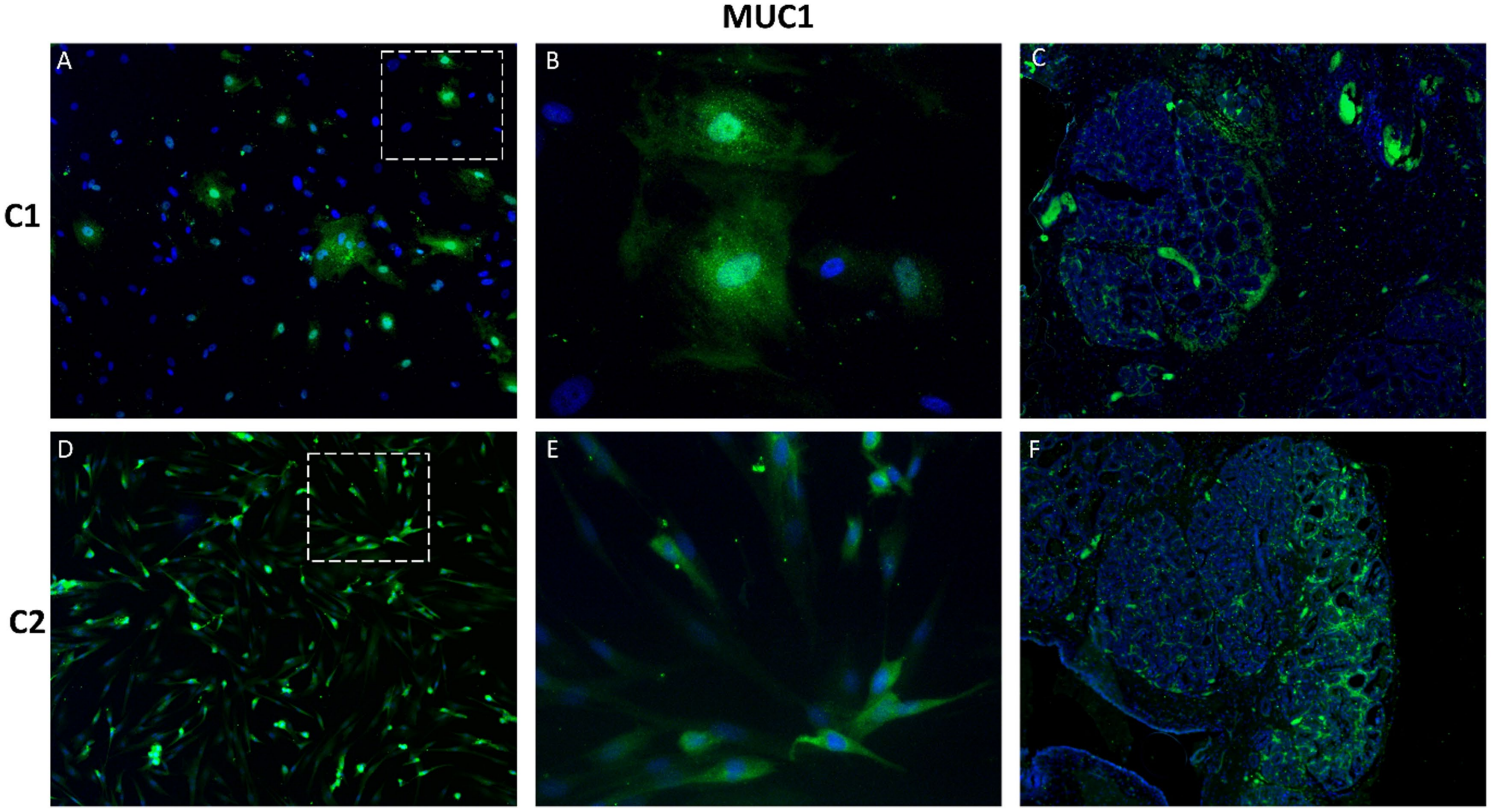
Immunofluorescence staining of MUC1 in primary canine mammary gland (CMG) cells and tissue. Representative images show strong MUC1 expression (green) with apical localization in cultured CMG cells **(A,B,D,E)** and corresponding tissue samples **(C,F)**. Nuclei are counterstained with DAPI (blue). Panels **B** and **E** represent details of **A** and **D**, respectively. Magnification: A, C, D, F = 100x; B, E = 400x.

MUC1, a transmembrane glycoprotein involved in epithelial cell protection and adhesion, was abundantly expressed in CMG cells, with apical localization consistent with non-malignant epithelial differentiation (Figure 7).

**Figure 8:**
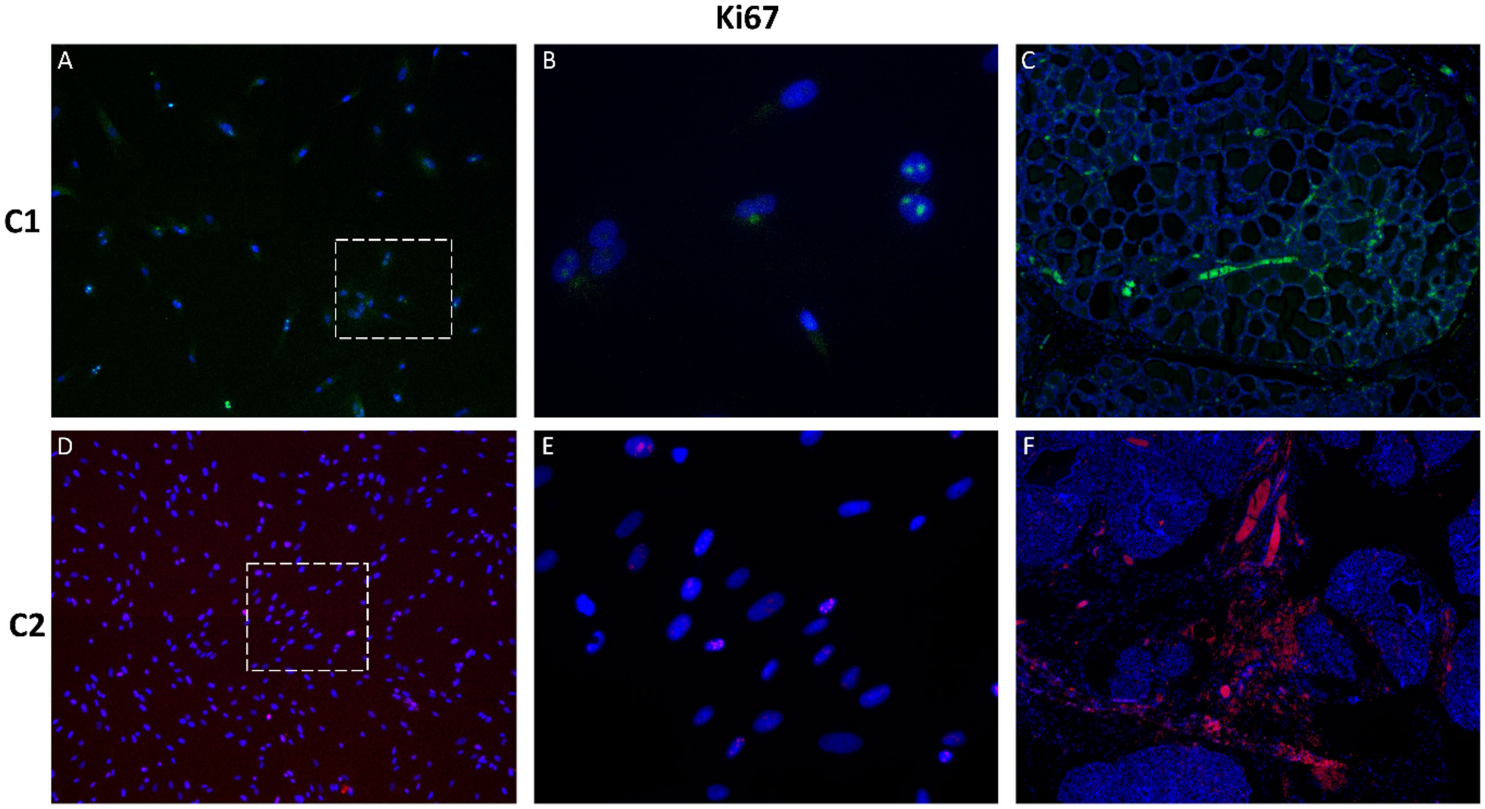
Immunofluorescence staining of the proliferation marker Ki-67 in primary CMG cells and tissue. Low levels of nuclear Ki-67 expression (red) were detected in cultured cells **(A,B,D,E)** and tissue samples **(C,F)**, indicating a moderate proliferative state. Nuclei were stained with DAPI (blue). Panels **B** and **E** show magnified regions of **A** and **D**, respectively. Magnification: A, C, D, F = 100x; B, E = 400x.

Analysis of the nuclear proliferation marker Ki-67 revealed low expression levels, indicating a moderate proliferative state in cultured CMG cells (Figure 8). This was consistent with their non-transformed phenotype and supports their relevance as a model for physiologically normal mammary epithelium.

Vimentin is an intermediate filament protein predominantly expressed in mesenchymal cell types. Fibroblasts generally display vimentin intermediate filaments (VIFs) evenly dispersed throughout their cytoplasm (12).

**Figure 9:**
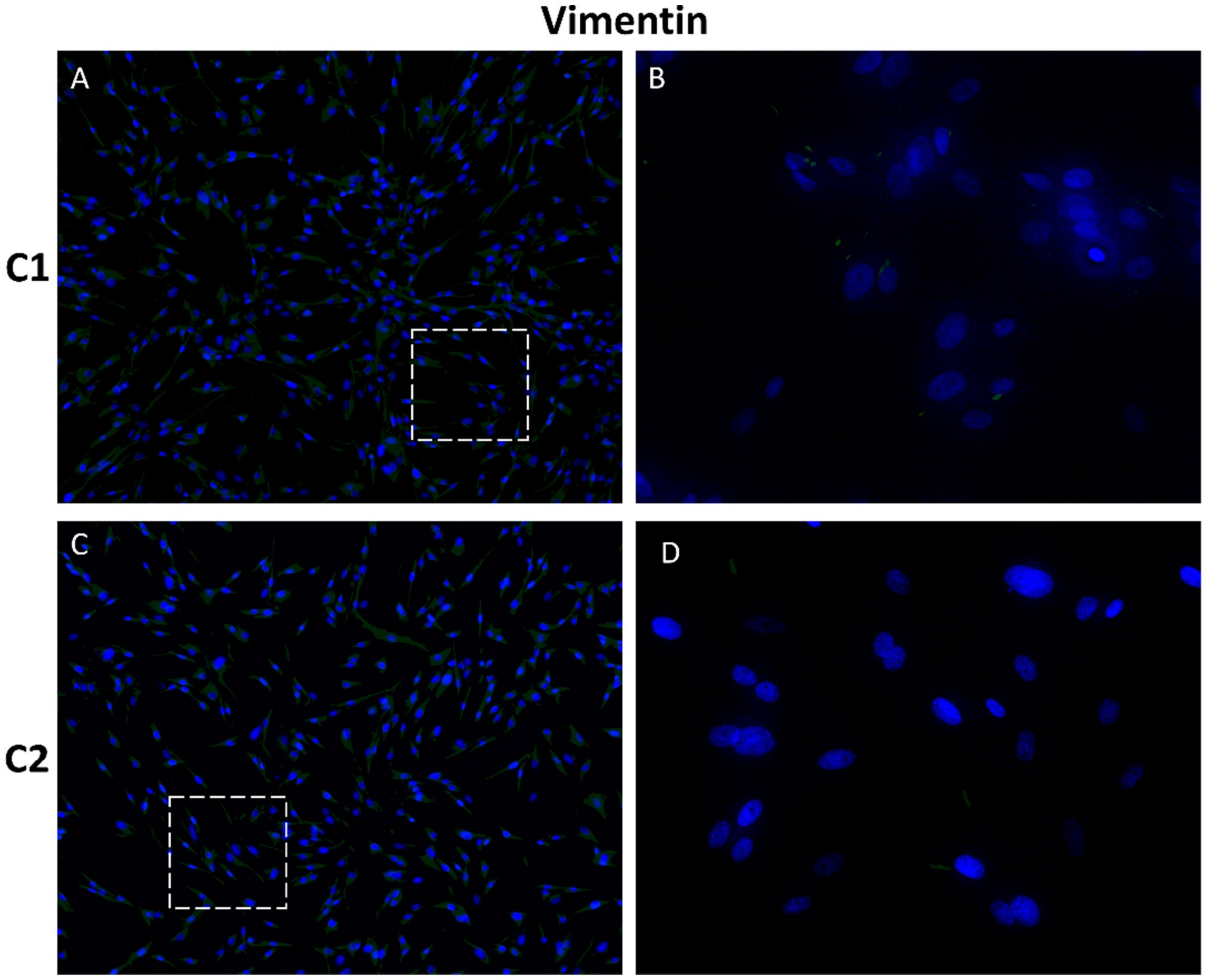
Immunofluorescence analysis of mesenchymal marker vimentin in primary CMG cells. Negative expression was observed in CMG cells **(A–D)** indicating non-mesenchymal potential of cells. Nuclei were stained with DAPI (blue). Panels **B** and **D** are details of **A** and **B**. Magnification: **(A)**, C = 100×; **(B)**, D = 400 ×.

**Figure 10:**
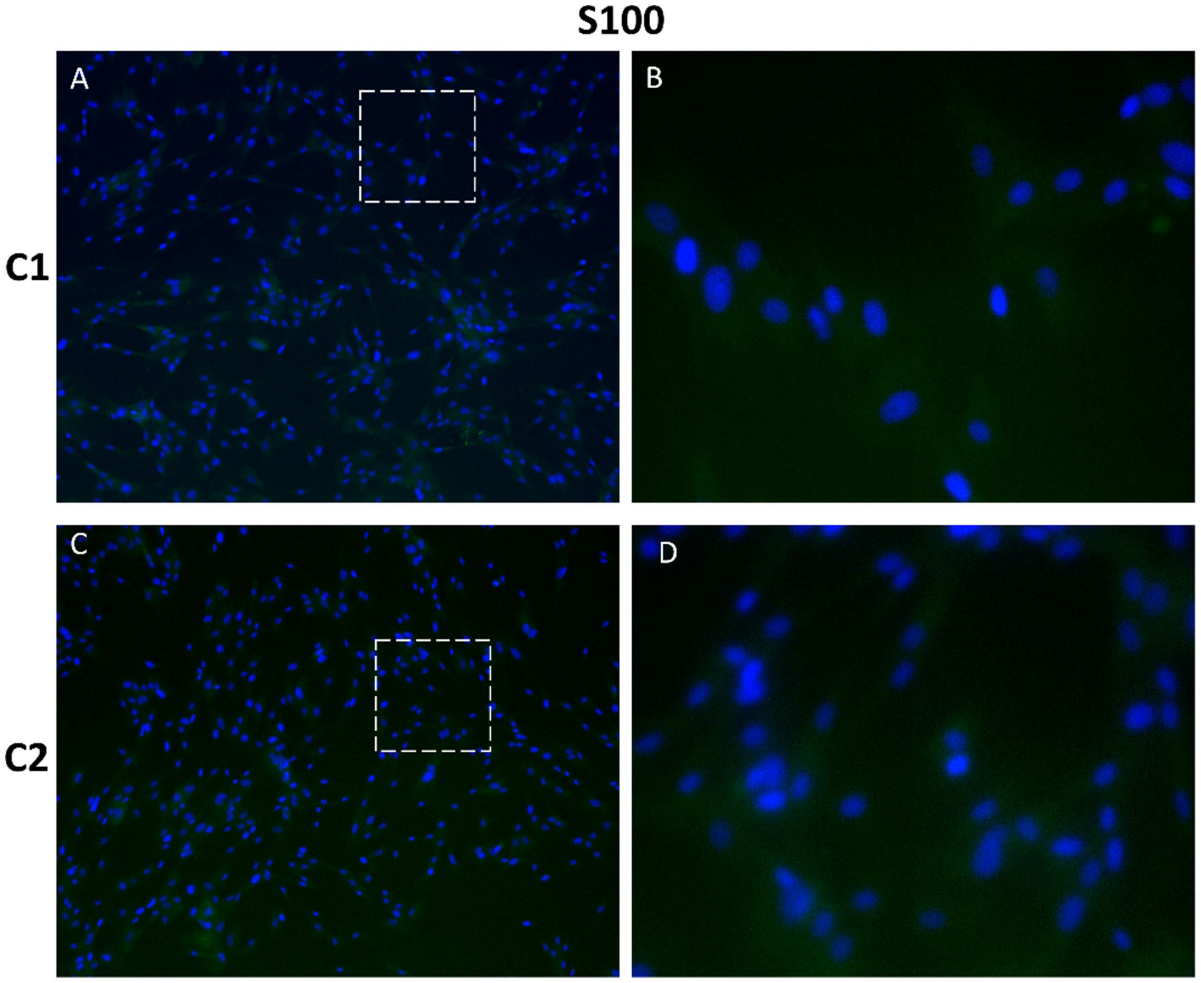
Immunofluorescence staining of protein S100 in primary canine mammary cells. Expression of marker S100 was negative in CMG cells **(A–D)**, indicating epithelial luminal origin of cells. Nuclei were counterstained with DAPI (blue). Panels **B** and **D** show magnified views of **A** and **C**, respectively. Magnification: **(A)**, C = 100×; **(B)**, E = 400 ×.

The S100 family comprises calcium-binding proteins involved in cell homeostasis, growth, proliferation, and differentiation, and is associated with cancer progression. S100 proteins are expressed in several cell types including fibroblasts, nerve cells and mammary myoepithelial cells (13–17). Negative expression of both mesenchymal markers, vimentin and S100, was observed in CMG cells (Figures 9, 10). Negative controls showed no specific staining for any of the investigated markers (Supplementary Figures S1–S3).

The immunofluorescence panel applied in this study (pan-CK, CK8/18, MUC1, Ki-67, vimentin and S100) consistently showed a luminal epithelial phenotype in cultured CMG cells. We did not detect a distinct cell population with morphological or immunophenotypic features compatible with myoepithelial cells, indicating that the established cultures are luminal-enriched epithelial monolayers.

## 4 Discussion

The mammary gland, a modified sweat gland, exhibits significant development in females and remains rudimentary in males (18). It is among the few mammalian tissues capable of undergoing cyclical morphological and functional changes - growth, differentiation, and regression - throughout the female’s reproductive life, particularly during puberty, pregnancy, lactation, and involution (19). This high degree of hormonal responsiveness renders mammary tissue particularly susceptible to neoplastic transformation (9, 20).

Mammary gland development initiates during embryogenesis with the formation of a rudimentary ductal tree embedded in the mammary fat pad (19). At the onset of puberty, ovarian hormones drive further ductal and alveolar development: estrogen promotes ductal elongation, while progesterone and prolactin orchestrate alveolar morphogenesis (11, 21). During pregnancy, adipose tissue is replaced by glandular epithelium capable of milk secretion. Following weaning, the gland undergoes involution, characterized by apoptosis and extracellular matrix remodeling (22).

Histologically, the active mammary gland comprises secretory alveoli and secretory tubules lined by cuboidal epithelium, supported by myoepithelial cells and a richly vascularized stroma. In contrast, the inactive gland post-lactation exhibits atrophy, with reduced parenchyma replaced by adipose tissue (23, 24). These phases were evident in our histological analysis (H&E): lactating tissue displayed well-developed alveolar structures, whereas tissue in regression showed disorganized, collapsed alveoli and expanded stromal components, aligning with previously published descriptions of the dynamic remodeling capacity of mammary tissue.

Canine mammary tumors (CMTs) are among the most common neoplasms in intact female dogs, accounting for a significant proportion of tumors in this population, with approximately half exhibiting malignant potential and metastatic behavior (25). Due to significant histopathological, hormonal, and molecular similarities with human BC, including shared oncogenic pathways and hormone responsiveness, CMTs serve as a valuable spontaneous model for translational BC research (26).

Historically, immortalized cell lines have been central to *in vitro* studies on CMTs. While these models offer consistency and scalability, they often fail to replicate the dynamic cellular heterogeneity and microenvironmental interactions of native tissues (27). Additionally, long-term culturing can induce genetic drift, reducing their translational fidelity and clinical relevance (28). These limitations have prompted a shift toward primary cell cultures derived directly from fresh tissue biopsies, which better retain *in vivo* phenotypic characteristics and patient-specific responses.

In our study, we successfully established a two-dimensional primary culture model from healthy CMG tissue using a combined mechanical and enzymatic dissociation protocol. This approach enabled efficient cell isolation, with cells demonstrating robust adherence, rapid proliferation, and confluence within 7 days (29). This methodological choice aligns with protocols employed in previous studies that utilized combined mechanical and enzymatic dissociation techniques to optimize cell yield and viability in canine mammary tissue (30–32). In contrast (33), reported the successful isolation of primary CMT cells using a mechanical-only method, although this approach may be less efficient for tissues with intact extracellular matrix or limited enzymatic susceptibility.

The efficacy of our combined dissociation method is further supported by the findings of Petroušková et al. (31), who established primary cell cultures from CMG tumors using mechanical disaggregation and enzymatic digestion with type IV collagenase. Their cultures achieved confluency by day 7 and exhibited high expression of epithelial markers along with significant proliferation activity. These results corroborate our observations of rapid cell proliferation and marker expression in primary CMG cultures. Regardless of the technique, published studies consistently report that isolated primary mammary cells - whether from tumor or normal tissue - retain the capacity for proliferation and *in vitro* propagation. Our findings corroborate this, as the CMG cells maintained their proliferative potential across passages, suggesting that our dissociation method preserved cellular integrity and stemness features relevant for subsequent experimentation.

Histological analysis of donor tissue revealed a well-organized lobular structure with active alveoli and ducts, lined by tall cuboidal epithelium and containing lactiferous secretions - features typical of lactating canine mammary glands (34). Similar architecture and epithelial marker expression were reported in tumor-derived cultures (31), supporting the tissue’s physiological integrity. By establishing a primary culture from non-neoplastic CMG tissue, we provide a relevant model for studying normal epithelial biology and early neoplastic changes. This model also offers a benchmark for assessing drug specificity and cytotoxicity, contributing to more accurate preclinical testing and personalized therapy development.

The establishment of primary cell cultures from healthy CMG tissue provides a physiologically relevant model for studying normal mammary function and early neoplastic transformation. To assess proliferative behavior and metabolic activity, we employed the xCELLigence system RTCA and the XTT assay, aiming to define optimal seeding densities for *in vitro* applications. RTCA, which continuously monitors cell proliferation, adhesion, and morphology through electrical impedance, revealed that primary CMG cells seeded at both 4,000 and 8,000 cells/well exhibited rapid early growth, with a clear plateau phase observed after 48 h. However, while 8,000 cells/well resulted in a sharp initial increase in CI due to rapid proliferation and early confluence (80–90%), this was followed by a decline in growth rate, likely driven by spatial constraints and contact inhibition. In contrast, cultures seeded at 4,000 cells/well displayed sustained and linear proliferation over a 24–72 h period, identifying this density as optimal for subsequent *in vitro* experimentation. These findings were corroborated by the XTT assay, which evaluates mitochondrial metabolic activity. CMG cells seeded at 8,000 cells/well showed high metabolic activity only during the first 48 h, after which activity declined, consistent with the plateau and growth inhibition observed in the xCELLigence data. Conversely, cells seeded at 4,000 cells/well maintained higher metabolic activity over a longer duration, supporting their continued viability and proliferative capacity. Together, these data point out the critical importance of optimizing initial seeding density in primary cell cultures to ensure that cells remain in the exponential growth phase. Such standardization enhances reproducibility and reliability in downstream biological and pharmacological assays, particularly in models intended for translational veterinary and comparative oncology research. Our findings align with previous studies using xCELLigence to track proliferation dynamics, such as de Oliveira et al. (51), who used the system to assess glycolysis inhibition in colorectal cancer cells. Likewise, the XTT assay remains a standard for evaluating mitochondrial activity and viability across diverse cell types. Importantly, this study is the first to define optimal seeding density for primary cultures derived from healthy mammary tissue in a 24-well format. In contrast, Petroušková et al. (33) used a lower density (1,000 cells/well) for CMT cultures, reflecting the higher proliferative capacity and altered phenotype of neoplastic cells, which also showed elevated expression of MUC1, CK8/18, and Ki-67. Optimizing growth conditions for healthy CMG cells offers a critical baseline for exploring mammary gland biology, hormonal regulation, and early tumorigenesis. This model also supports the assessment of drug effects on non-malignant cells, key for enhancing therapeutic specificity and safety (35). Ultimately, such cultures contribute to the goals of personalized medicine by replicating normal tissue physiology and advancing both basic and translational veterinary oncology research.

Our immunofluorescence analysis of primary CMG cells revealed robust expression of pan-CK and CK8/18, affirming their epithelial identity (36). Pan-CK antibodies, such as AE1/AE3, target a broad spectrum of cytokeratins, making them effective markers for epithelial cells in both canine and human tissues (37). CK8 and CK18 are intermediate filament proteins characteristic of luminal mammary epithelial cells, and their strong expression in CMG cultures mirrors their localization in normal human breast tissue, indicating a well-differentiated epithelial phenotype (38, 39). In human BC, the expression patterns of CK8/18 are associated with tumor subtypes and prognoses. Retention of CK8/18 expression correlates with luminal A and B subtypes, which generally have favorable outcomes. Conversely, loss or mislocalization of CK8/18 is linked to basal-like, more aggressive phenotypes (39, 40). Similarly, in canine mammary tumors, benign lesions typically maintain CK8/18 expression, while malignant tumors often exhibit reduced levels, underscoring the role of cytokeratin patterns as indicators of differentiation status (7, 41). However, cytokeratin profiling alone does not provide comprehensive information about proliferative or mesenchymal features. Co-staining with markers such as Ki-67 for proliferation or vimentin for mesenchymal characteristics is necessary to fully characterize growth dynamics and epithelial-mesenchymal transition (42). Moreover, variability in cytokeratin expression due to tissue origin, culture conditions, and passage number highlights the need for standardized protocols to ensure reproducible phenotyping across laboratories (43, 44). Overall, the observed pan-CK and CK8/18 positivity in primary CMG cells aligns with established patterns in human and canine mammary tissues, validating these cultures as models for studying normal epithelial biology and early tumorigenic events (45).

Immunofluorescence analysis of primary CMG cells in our study revealed strong expression of MUC1, a transmembrane glycoprotein integral to epithelial protection and signaling. In normal mammary epithelium, MUC1 is predominantly localized to the apical surface, contributing to the maintenance of cell polarity and forming a protective barrier - an indicator of a well-differentiated state (46, 47). In malignancy, however, MUC1 expression becomes dysregulated. In human BC, MUC1 often shifts from apical to diffuse cytoplasmic or circumferential localization, correlating with loss of cell polarity, invasiveness, and poor prognosis (48, 49). Aberrant glycosylation further enhances MUC1’s pro-tumorigenic role, aiding in immune evasion and metastasis (50). Comparable patterns are observed in canine mammary tumors, where normal tissues exhibit apical MUC1 expression, while malignant ones display cytoplasmic redistribution, linked to higher tumor grade and metastatic behavior (51). In our study, CMG cells retained apical MUC1 localization consistent with non-malignant mammary epithelium, highlighting their potential as a pre-neoplastic *in vitro* model. This preserved epithelial phenotype makes them well-suited for investigating early alterations in MUC1 expression and function that may contribute to tumor initiation. Moreover, CMG cultures represent a promising platform for studying the mechanisms of MUC1 dysregulation in cancer progression and for preclinical evaluation of MUC1-targeted therapeutic strategies.

Immunofluorescence staining showed minimal Ki-67 positivity in primary CMG cells, reflecting limited proliferative activity and a predominantly quiescent cellular phenotype. Our finding aligns with the expected profile of non-malignant mammary epithelium, where Ki-67 levels are typically low due to limited mitotic activity. In contrast, elevated Ki-67 expression is a hallmark of CMTs, reflecting their higher proliferative capacity. For instance, Carvalho et al. (52) reported Ki-67 indices ranging from 3 to 32% in benign tumors and 20–49% in malignant ones, correlating with tumor aggressiveness and prognosis. Similarly, in human BC, high Ki-67 expression is associated with increased tumor grade, shorter survival, and higher recurrence risk. A meta-analysis by de Azambuja et al. (53) confirmed its prognostic value in early-stage BC, demonstrating that Ki-67 positivity confers a higher risk of relapse and worse survival. Furthermore, Luporsi et al. (54) emphasized the predictive relevance of Ki-67 for chemotherapy response, noting that high Ki-67 levels are associated with increased pathological complete response rates in patients undergoing neoadjuvant chemotherapy. The retention of low Ki-67 expression in CMG cultures underscores their utility as a model for studying normal proliferation and early neoplastic changes in both canine and human mammary biology. These cultures provide a relevant platform for investigating the mechanisms underlying Ki-67 dysregulation and its role in tumor progression, offering insights applicable to both veterinary and human oncology.

Immunofluorescence analysis of mesenchymal markers, vimentin and S100, showed negativity for both markers in CMG cells, indicating an epithelial phenotype. Vimentin is typically expressed in cells of mesenchymal origin and regulates cell growth, migration and the maintenance of cellular integrity (12, 55). It has also been associated with cancer invasiveness and increased expression of vimentin was observed in various tumor cells and is considered a marker of epithelial-mesenchymal transition (56, 57). Moreover, vimentin is expressed in the stromal and myoepithelial cell population of the mammary gland (16, 17). The S100 family has many isoforms, and their expression varies by tissue and condition; S100 proteins can be found in fibroblasts, astrocytes, melanocytes, and myoepithelial cells, among others (15, 58–61). Overexpression of some members of S100 family has been reported in several common cancers, including breast cancer (14).

While our findings confirm the epithelial nature and low proliferative activity of CMG cells, certain limitations should be acknowledged. Marker expression was evaluated qualitatively; quantitative techniques such as RNA *in situ* hybridization, as demonstrated by Li et al. (62) for HER2 mRNA in canine mammary tumors, could provide more precise molecular profiling. Our marker panel was limited; inclusion of basal and myoepithelial markers (e.g., CK5, CK14, *α*-SMA, p63) would allow deeper insight into CMG cell heterogeneity, as these markers are key in distinguishing myoepithelial populations involved in tumor progression (63–65). In the present work, we deliberately focused on establishing a reference luminal epithelial culture from normal CMG tissue and therefore selected a restricted immunofluorescence panel targeting epithelial and luminal markers (pan-CK, CK8/18, MUC1) together with Ki-67 and selected mesenchymal markers (vimentin, S100). Basal/myoepithelial markers (e.g., CK5, CK14, α-SMA, p63) were not included because the available primary material had to be prioritized for initial luminal characterization. Moreover, the dissociation protocol and standard 2D culture conditions used here favor luminal epithelial outgrowth, whereas myoepithelial cells typically require selective enrichment and tailored media to be maintained *in vitro* (29, 65, 66). In line with this, our cultures showed a homogeneous luminal phenotype, and we did not observe an expanding myoepithelial compartment, suggesting that myoepithelial cells from the original tissue did not efficiently proliferate or were gradually lost during early passages. Additionally, the role of MUC1 in normal versus malignant contexts requires further study. While apically localized in healthy tissue, MUC1 becomes mislocalized and aberrantly glycosylated in tumors, contributing to metastasis and immune evasion. Clarifying these functional shifts could enhance our understanding of mammary gland pathophysiology (67). Future work addressing these aspects will improve the value of CMG cultures as models for mammary biology and tumorigenesis.

## 5 Conclusion

This study is the first to successfully establish and characterize primary epithelial cell cultures directly from histologically normal CMG tissue, providing a relevant *in vitro* model for studying normal mammary physiology, early tumorigenesis, and supporting future development of standardized control cell lines. Histological analysis confirmed lobular structures typical of actively lactating tissue, validating the functional state of the donor gland. CMG cells adhered efficiently, reached confluence within 7 days, and showed optimal proliferation at 4,000 cells/well, as verified by xCELLigence and XTT assays. These standardized culture conditions offer a practical reference for veterinary laboratories working in diagnostic, pharmacological, or oncological research. Immunofluorescence analysis confirmed epithelial identity (pan-CK, CK8/18), low Ki-67 expression, negative expression of mesenchymal markers (vimentin, S100) and apical MUC1 localization - features indicative of a non-malignant, differentiated phenotype. These data support the use of CMG cultures as a baseline model for distinguishing physiological epithelial characteristics from tumor-associated changes in canine mammary tissue. This model contributes to comparative oncology by enabling more accurate evaluation of tumor biology and therapeutic responses. However, limitations include the qualitative nature of marker assessment and the restricted panel used. Future work should apply quantitative techniques and include additional markers (CK5, CK14, *α*-SMA, p63), as well as investigate MUC1’s role in canine mammary pathology. Overall, CMG cultures provide a valuable platform for translational research and the development of personalized veterinary therapies.

## Data Availability

The original contributions presented in the study are included in the article/Supplementary material, further inquiries can be directed to the corresponding author.
