# Supplementary material for: Establishment and characterization of primary epithelial cell cultures from healthy canine mammary gland tissue
Source: Front Vet Sci. 2026 Jan 28;12:1652991. doi: 10.3389/fvets.2025.1652991 (PMC12890635; doi:10.3389/fvets.2025.1652991)
Supplement: Supplementary file 1 [file Data_Sheet_1.docx]

Supplementary Material: Establishment and Characterization of Primary Epithelial Cell Cultures from Healthy Canine Mammary Gland Tissue


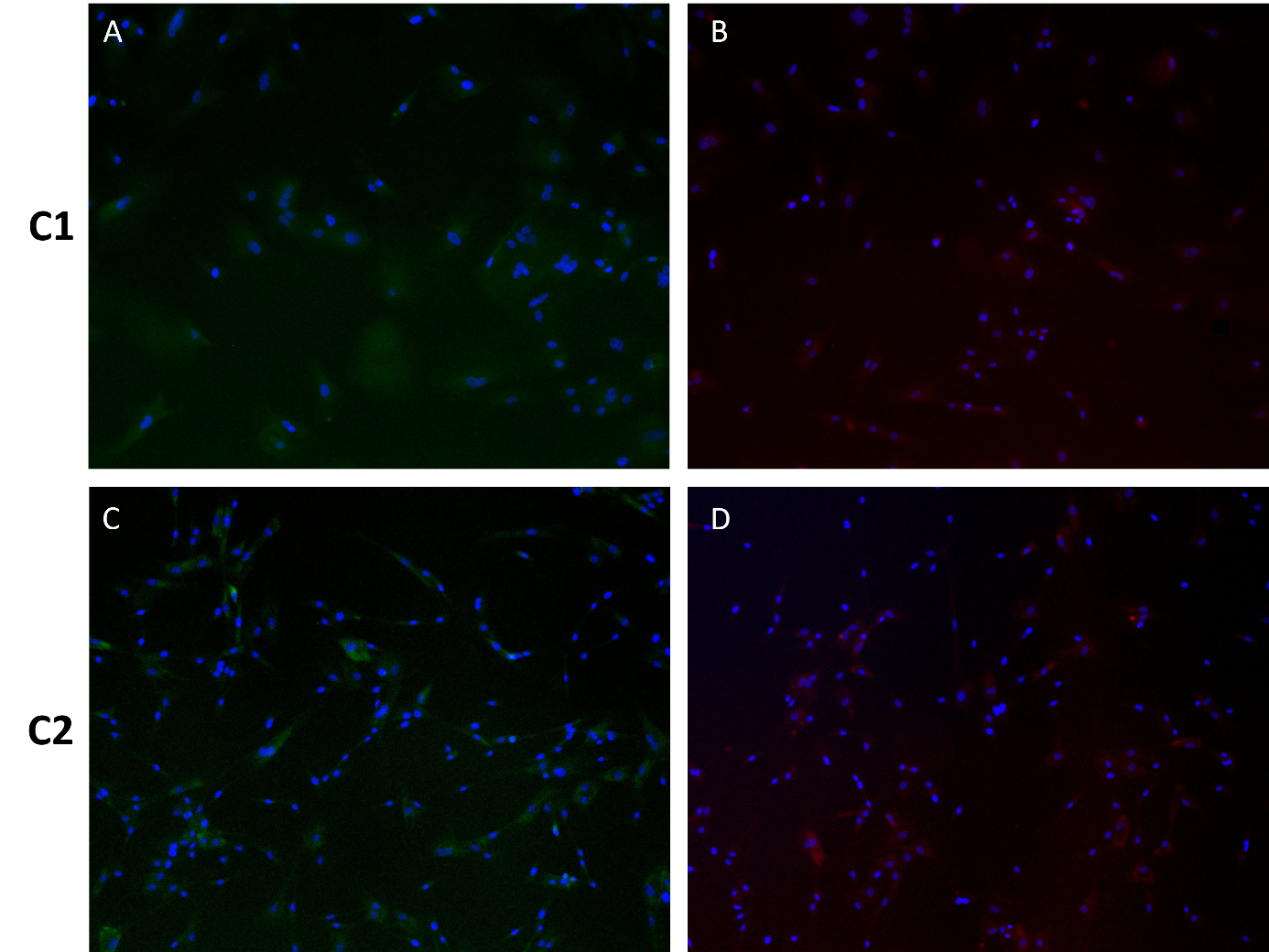


Supplementary Figure S1. Immunocytochemical analysis of negative controls in CMG cells, where primary antibodies (green - A, C; red – B, D) were excluded from the assay. Nuclei were stained with DAPI. Magnification: 100x.


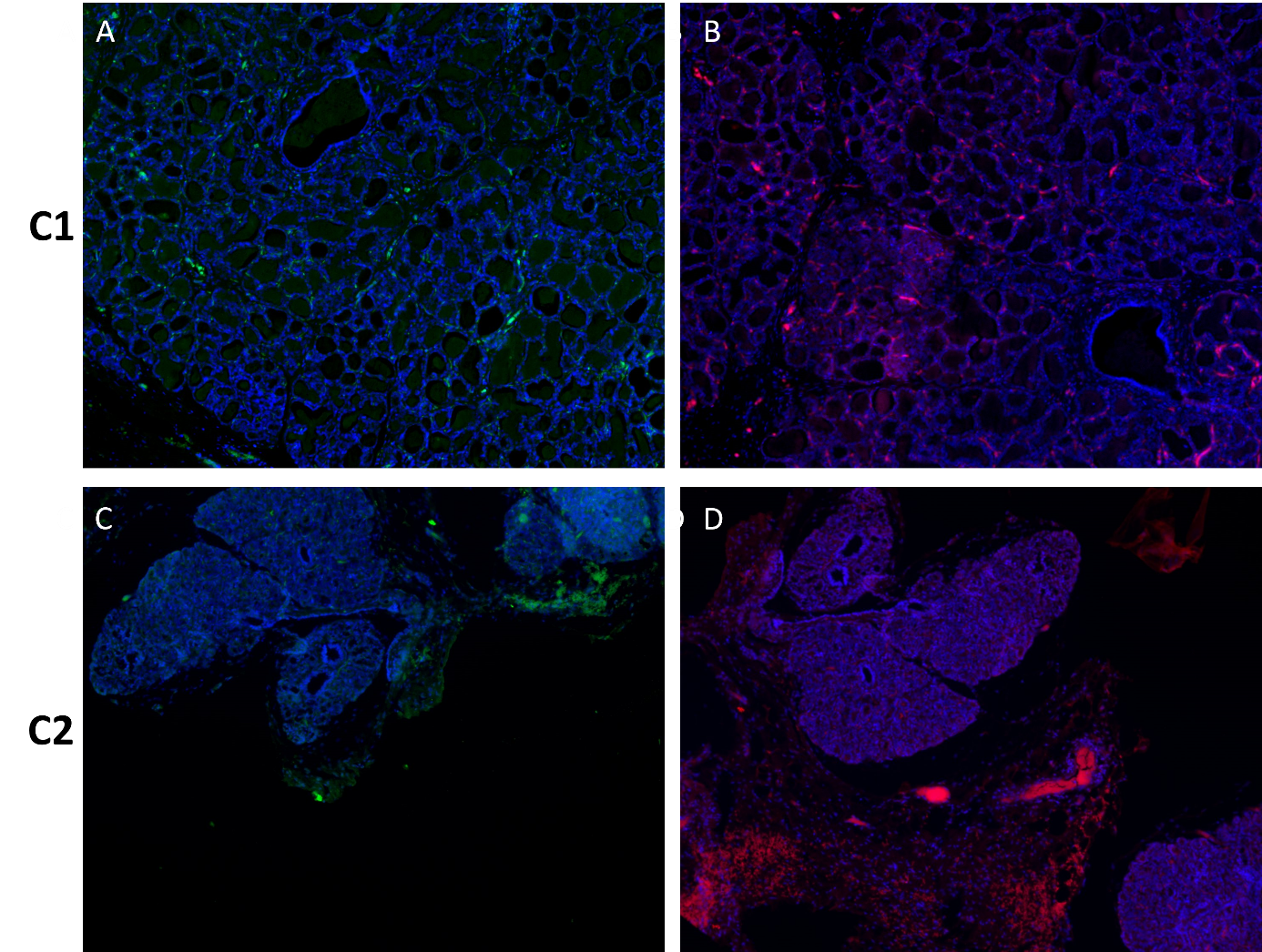


Supplementary Figure S2. Immunohistochemical analysis of negative controls in CMG tissues, where primary antibodies (green - A, C; red – B, D) were excluded from the assay. Nuclei were stained with DAPI. Magnification: 100x.


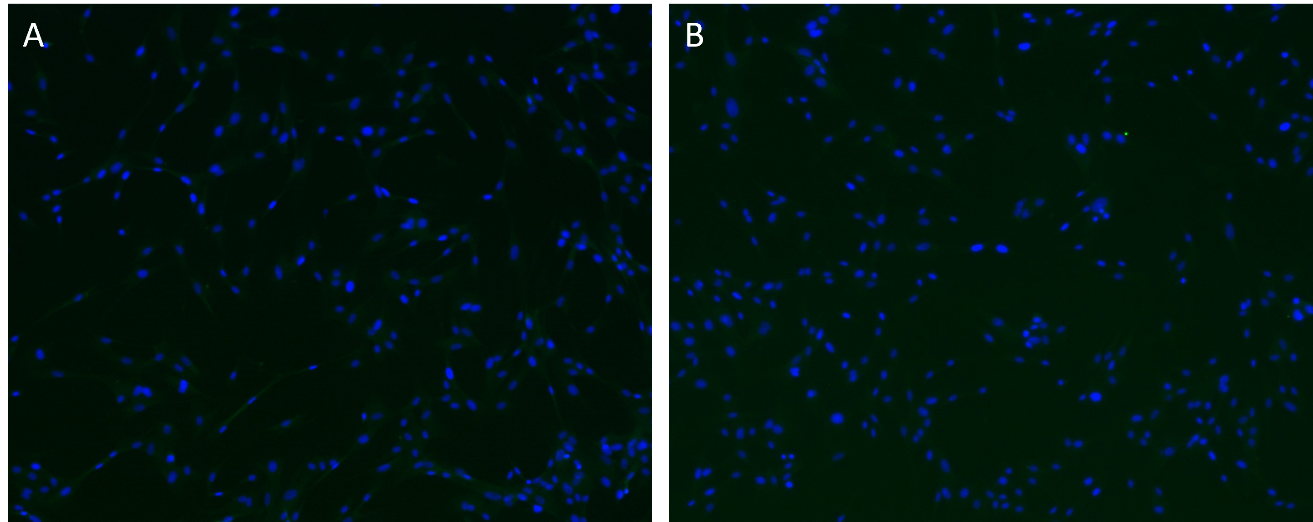


Supplementary Figure S3. Immunocytochemical analysis of negative controls in CMG cells C1 (A) and C2 (B), where primary antibodies (green) were excluded from the assay. Nuclei were stained with DAPI. Magnification: 100x.
